# A multimodal noninvasive strategy for enhanced early diagnosis of patient with type B chronic gastritis infected with *Helicobacter pylori*

**DOI:** 10.1097/MD.0000000000049676

**Published:** 2026-07-10

**Authors:** Yin Xiong, Beibei Wang, Ke Li, Xin Chen, Ping Wu, Wei Huang

**Affiliations:** a Department of Laboratory Medicine, Wuhan No. 1 Hospital, Wuhan, Hubei, PR China; b College of Traditional Chinese Medicine, Hubei University of Chinese Medicine, Wuhan, Hubei, PR China; c Department of Pathophysiology, Tongji Medical College, Huazhong University of Science and Technology, Wuhan, Hubei, China.

**Keywords:** ^13^C-UBT, chronic atrophic gastritis, chronic non-atrophic gastritis, CIM test, *H pylori* antibody serotyping

## Abstract

This study aims to assess the diagnostic efficacy of *Helicobacter pylori* antibody serotyping, current infection marker (CIM) testing, and urea breath test (UBT), both individually and in combination, for detecting *H pylori* infection in patients with type B chronic gastritis. A total of 159 subjects were enrolled for detecting the levels of *H pylori* antibody serotyping using enzyme-linked immunosorbent assay, anti-CIM immunonglobulin G antibody via immunochromatographic assay, and ^13^C-UBT using an infrared spectrometer. The results revealed that levels of *H pylori* serotyping antibodies and ^13^C-UBT were significantly elevated in patients with chronic non-atrophic gastritis and chronic atrophic gastritis compared to those in healthy controls, with a strong positive correlation between these markers (*P* < .05). Positive rates for CIM and type I *H pylori* serotyping were significantly higher in the type B chronic gastritis group than in healthy individuals (*P* < .05). A high positive coincidence rate was exhibited among the 3 tests. Notably, the simultaneous positivity or negativity of all 3 tests was highly consistent with the gastroscopic diagnosis of chronic gastritis or a healthy status, respectively, and this strong concordance highlighted the diagnostic value of the combined test results. In addition, the 3-test combination provided superior diagnostic value in sensitivity and accuracy than any single test or 2-test combination, achieving the highest area under the curve of 0.952. These findings suggest that the combined application of the 3 tests can serve as a noninvasive early screening diagnostic indicator for chronic non-atrophic gastritis and chronic atrophic gastritis, which may contribute to improving early clinical prevention and treatment.

## 1. Introduction

Gastritis, an inflammatory disease associated with gastric mucosa injury, is classified as either acute or chronic active gastritis.^[1]^ The progression of gastritis to gastric cancer is a gradual process, evolving from chronic non-atrophic gastritis (CNAG) to chronic atrophic gastritis (CAG), followed by intestinal metaplasia, dysplasia, and ultimately carcinoma. Chronic active gastritis, which includes autoimmune gastritis (type A) and infectious gastritis (type B), represents the initial and crucial stages of gastritis progression. Importantly, type B chronic gastritis, due to concentrated *Helicobacter pylori* infection in the stomach and duodenal bulbs, manifests as CNAG or CAG. The virulence factors contribute to a complex inflammatory response, consequently driving the development of gastric mucosal atrophy and erosion.^[2,3]^

*H pylori* infection exhibits a characteristic global distribution marked by substantial geographical variations.^[4]^ The prevalence is notably higher in less industrialized countries than in developed nations. Additionally, the incidence of *H pylori* infection has gradually declined alongside improvements in living standards and implementation of eradication strategies.^[5]^ Most individuals in the initial stages of *H pylori* infection are either asymptomatic or experience mild dyspepsia. However, persistent infection can lead to severe complications including peptic ulcers, mucosa-associated lymphoid tissue lymphoma, and gastric adenocarcinomas.^[6]^

Early diagnosis of *H pylori* infection is crucial. The diagnostic methods for *H pylori* infection can be categorized into invasive techniques, such as the rapid urease test, histology, and culture from biopsy samples obtained via gastroscopy, and noninvasive techniques, including serological antibody-based tests, urea breath tests (UBT), and stool antigen tests.^[5,7]^ Although the UBT is widely used as a noninvasive diagnostic method, it presents several challenges, including high costs and the need for skilled technicians to ensure accurate interpretation.^[8]^ Increasing evidence indicates the importance of identifying novel indicators for diagnosing and determining the type of *H pylori* infection. Tests, such as *H pylori* serotyping and the serological current infection marker (CIM) test, can also identify active *H pylori* infections.^[8–10]^ Therefore, the aim of the present study was to evaluate the clinical diagnostic value of *H pylori* antibody serotyping, serological CIM, and UBTs, both individually and in combination, for detecting *H pylori* infection in cases of CNAG and CAG and to determine whether these indicators could serve as noninvasive early screening diagnostic tools for CNAG and CAG, as alternatives to endoscopic biopsy, through the combined application of these tests.

## 2. Materials and methods

### 2.1. Patients

A total of 159 participants (60 healthy controls, 47 patients with CNAG, and 52 patients with CAG) from Wuhan No.1 Hospital were enrolled between March 2024 and September 2024. The diagnosis of chronic gastritis was based on gastrointestinal endoscopy and histopathological findings. Subjects with underlying diseases such as other tumors, primary and secondary severe organ damage, a history of stomach resection, prior *H pylori* therapy, and recent use of antibiotics or antisecretory drugs within the last 4 weeks were excluded. Blood samples were collected and sera were isolated by centrifugation until analysis. The study protocol was approved by the Ethics Committee of Wuhan No.1 Hospital.

### 2.2. Measurement of *H pylori* antibody serotyping

The double-antigen sandwich enzyme-linked immunosorbent assay was used to detect the serum concentrations of anti-cytotoxicity-associated gene A (CagA) antibody, anti-vacuolating cytotoxin A (VacA) antibody, and anti-Urease antibody produced by *H pylori* infection with supporting reagents using the AFS3000 B multichannel dry fluorescent immune analyzer (Chongqing iSIA Bio-technology Co., Ltd), which is a quantitative method. When the anti-Urease antibody was positive, while anti-CagA and anti-VacA antibodies were negative, the strains of infection were type II *H pylori* which are weakly toxic or nontoxic. When the anti-Urease antibody was positive, anti-CagA antibody or/and anti-VacA antibody were positive, and the strains of infection were type I *H pylori* with strong toxicity. Tests for 3 anti-Urease, anti-CagA, and anti-VacA antibodies were negative, indicating the absence of *H pylori* infection.

### 2.3. Detection of current *H pylori* infection (CIM test)

The indirect solid-phase immunochromatography assay was applied to detect *H pylori*-specific immunoglobulin G (IgG) antibodies and anti-CIM IgG antibodies using the MP Diagnostics ASSURE *H pylori* Rapid Test (MP Biomedicals Asia Pacific Pte Ltd). The presence of anti-CIM IgG antibodies indicated an ongoing *H pylori* infection. Serum specimens were applied to a designated area containing the immobilized *H pylori* antigens. A buffer was subsequently added to this area, facilitating the reaction between the colloidal gold-labeled antihuman IgG conjugate and the antigen-antibody complex as the samples reached the indicator line. The results were observed after 15 minutes. The test displayed 3 bands: band A served as the quality control line, band B indicated the CIM-positive line, and band C represented the line for *H pylori*-specific IgG antibodies. If both bands B and C were positive, it suggested that *H pylori* infection was ongoing. Conversely, if band B was negative and band C was positive, it indicated that *H pylori* infection had occurred but was not currently active.

### 2.4. Detection of ^13^C-UBT

The ^13^C-UBT was conducted to detect *H pylori* infection. The subjects were requested to fast for at least 3 hours prior to the collection of the baseline sample. Subsequently, a ^13^C urea tablet was ingested with warm water, and a test sample was collected 30 minutes later. Both the 0-minute and 30-minute samples were analyzed using an external ^13^C infrared spectrometer for the detection of ^13^CO_2_ (HCBT-01 respirometer, Shenzhen Zhonghe Haidwei Biotechnology Co., LTD). The detection value, referred to as the delta over baseline (DOB), was calculated using the formula DOB = (testing value − base value)/ base value × 100%. A positive result for *H pylori* infection was established when the DOB was equal to or > 4.0.

### 2.5. Statistical analysis

All statistical analyses were conducted using SPSS software version 31.0 (IBM Crop). Quantitative data that followed a normal distribution are presented as mean ± standard deviation (x ± s), while data with a non-normal distribution are reported as median ± interquartile range. An unpaired Student *t*-test was used for comparisons between 2 independent groups with normally distributed data, whereas the Mann–Whitney *U* test was used for non-normally distributed data. For comparisons involving more than 2 groups, 1-way analysis of variance was applied to normally distributed data, and the Kruskal–Wallis *H*-test was used for non-normally distributed data. Categorical qualitative data were analyzed using the chi-squared test for intergroup comparisons. The correlation between anti-urease antibody, anti-CagA antibody, anti-VacA antibody, and ^13^C-UBT was determined using pairwise Spearman correlation analysis. The diagnostic values of the selected parameters were evaluated using receiver operating characteristic (ROC) curve analysis, and the area under the ROC curve (AUC) was calculated.

## 3. Results

### 3.1. Elevated serum levels of *H pylori* antibody serotyping in patients with CNAG and CAG

**Figure 1.**
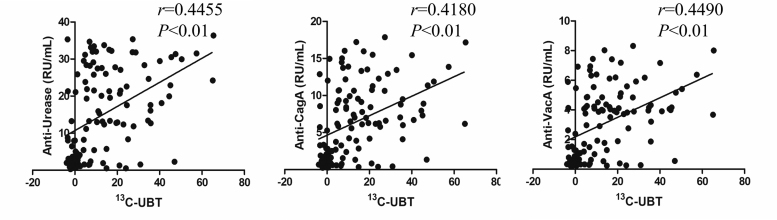
The correlation analysis between anti-Urease antibody, anti-CagA antibody, anti-VacA antibody and ^13^C-UBT in CNAG and CAG groups were analyzed using pairwise Spearman correlation analysis. The correlation was represented by biomarker-biomarker correlation (*r*); *R* > 0 indicated a positive correlation, and *R* < 0 indicated a negative correlation. All correlations shown in this figure had a statistical difference, *P* < .05. CAG = chronic atrophic gastritis, CagA = cytotoxicity-associated gene A, CNAG = chronic non-atrophic gastritis, UBT = urea breath test, VacA = vacuolating cytotoxin A.

The characteristics of the 159 subjects were divided into 3 groups: 60 healthy controls, 47 patients with CNAG, and 52 patients with CAG (Table 1). The age and sex distributions of all patients were similar to those of the healthy controls. Serum levels of anti-Urease, anti-CagA, anti-VacA IgG, and ^13^C-UBT were significantly elevated in both the CNAG and CAG groups compared to those in the healthy controls (*P* < .05) (Table 1). Notably, the levels of anti-Urease, anti-CagA, and anti-VacA antibodies were lower in the CAG group than in the CNAG group; however, the difference was not statistically significant. Correlation analysis revealed that ^13^C-UBT levels in the CNAG and CAG groups were positively correlated with the antibody levels of anti-Urease (*R* = 0.4455), anti-CagA (*R* = 0.4180), and anti-VacA (*R* = 0.4490) (Fig. 1), with significant differences (*P* < .01). These findings suggest that elevated levels of *H pylori* antibody serotyping and ^13^C-UBT are closely associated with the clinical diagnosis and progression of *H pylori*-related type B chronic gastritis.

**Table 1:** Characteristics of the study subjects and *H pylori* antibody serotyping findings.

Characteristics	HC	CNAG group	CAG group
Number	60	47	52
Age (yrs)	60 (45–79)	59 (47-75)	62 (47-74)
Gender (n, male)	36	27	29
^13^C-UBT	0.7 (3.5)	14.75 (19.7)*	9.05 (21.15)†
anti-Urease (RU/mL)	2.98 ± 3.08	18.48 ± 11.38*	16.83 ± 10.87†
anti-CagA (RU/mL)	1.55 ± 1.87	7.91 ± 4.96*	7.20 ± 4.67†
anti-VacA (RU/mL)	0.71 ± 0.82	3.83 ± 2.26*	3.44 ± 2.16†

Data were described as median (range), median (interquartile range), or mean ± SD unless otherwise stated.

CAG = chronic atrophic gastritis, CagA = cytotoxicity-associated gene A, CIM = current infection marker, CNAG = chronic non-atrophic gastritis, HC = healthy controls, SD = standard deviation, UBT = urea breath test, VacA = vacuolating cytotoxin A.

* *P* < .05 for patients with CNAG compared with healthy controls.

† *P* < .05 for patients with CAG compared with healthy controls.

### 3.2. Elevated positive rates of serological CIM antibody and *H pylori* serotyping antibody in the diagnosis of CAG and CNAG

The positivity rates for anti-CIM IgG antibody and *H pylori* antibody serotyping are presented in Table 2. Compared to healthy controls, the positive rate of anti-CIM IgG antibody was significantly elevated in both the CNAG and CAG groups (*χ*^2^ = 36.84, *P* < .01; *χ*^2^ = 39.42, *P* < .01, respectively). However, no significant difference was observed between the CNAG and CAG groups. Additionally, the positivity rate for Type I *H pylori* was markedly higher than that for Type II *H pylori* in both the CNAG and CAG groups. The positivity rate of *H pylori* antibody serotyping was also significantly higher in the CNAG and CAG groups than in the healthy controls (*χ*^2^ = 53.58, *P* < .01; *χ*^2^ = 46.67, *P* < .01, respectively). Notably, some healthy controls were positive for anti-CIM IgG antibodies or *H pylori* antibody serotyping, and anti-CIM IgG antibody or *H pylori* antibody serotyping was not detected in a minority of the CNAG and CAG groups with *H pylori* infection. This finding indicates the limitations of relying solely on individual methods for the CIM test or *H pylori* antibody serotyping test.

**Table 2:** The positive rate of CIM test and *H pylori antibody* serotyping test in patients with chronic gastritis by *H pylori* infection.

Subjects	CIM test			*H pylori* antibody serotyping test			
	Positive	Negative	*X* ^2^	Type I *H pylori*	Type II *H pylori*	Negative	*X* ^2^
	n (%)	n (%)		n (%)	n (%)	n (%)	
HC	5 (8.3)	55 (91.7)		4 (6.7)	2 (3.3)	54 (90)	
CNAG group	31 (66)	16 (34)	36.84*	29 (61.8)	9 (19.1)	9 (19.1)	53.58^a^
CAG group	35 (67.3)	17 (32.7)	39.42†; 0.02‡	32 (61.5)	6 (11.5)	14 (27)	46.67^b^;0.74^c^

Data were described as number (percent).

CAG = chronic atrophic gastritis, CIM = current infection marker, CNAG = chronic non-atrophic gastritis, HC = healthy controls, n = number of patients.

* *P* < 0.01 for patients with CNAG compared with healthy controls.

† *P* < .01 for patients with CAG compared with healthy controls.

‡ *P* > .05 for patients with CAG compared with CNAG.

### 3.3. High positive coincidence rates among the *H pylori* antibody serotyping test, CIM test, and UBT test in patients with type B chronic gastritis groups and healthy controls

The comparison of positive and negative rates between the *H pylori* antibody serotyping test, CIM test, and ^13^C-UBT is presented in Table 3. The results showed that subjects with a positive ^13^C-UBT were more likely to be infected with type I *H pylori* strains and less likely to be infected with type II *H pylori* strains. Some subjects with a negative ^13^C-UBT were positive for *H pylori* antibody serotyping or anti-CIM IgG antibody, and some subjects with a positive ^13^C-UBT were negative for *H pylori* antibody serotyping or anti-CIM IgG antibody, which further illustrates the limitation of a single methodology. There were 76 subjects with simultaneous positivity between *H pylori* antibody serotyping and UBT tests, with a positive coincidence rate of 85.39% in reference to the ^13^C-UBT test, while there were 64 subjects with simultaneous negativity, and the negative coincidence rate was up to 91.40%. Furthermore, there were 66 subjects with simultaneous positivity between the CIM test and ^13^C-UBT test; the positive coincidence rate was 74.16% in reference to the ^13^C-UBT test, and there were 65 subjects with simultaneous negativity, and the negative coincidence rate was notably higher at 92.86%.

**Table 3:** Comparison of the positive rates between *H pylori* antibody serotyping test or CIM test and UBT test.

^13^C-UBT	*H pylori* antibody serotyping test			CIM test	
	Type I *H pylori* (n)	Type II *H pylori* (n)	Negative (n)	Positive (n)	Negative (n)
Positive	62	14	13	66	23
Negative	3	3	64	5	65
Positive coincidence rate (%)	85.39			74.16	
Negative coincidence rate (%)	91.40			92.86	

Data were described as number and percent. The positive and negative coincidence rates were in reference to the ^13^C-UBT test.

CIM = current infection marker, n = number of patients, UBT = urea breath test.

**Figure 2.**
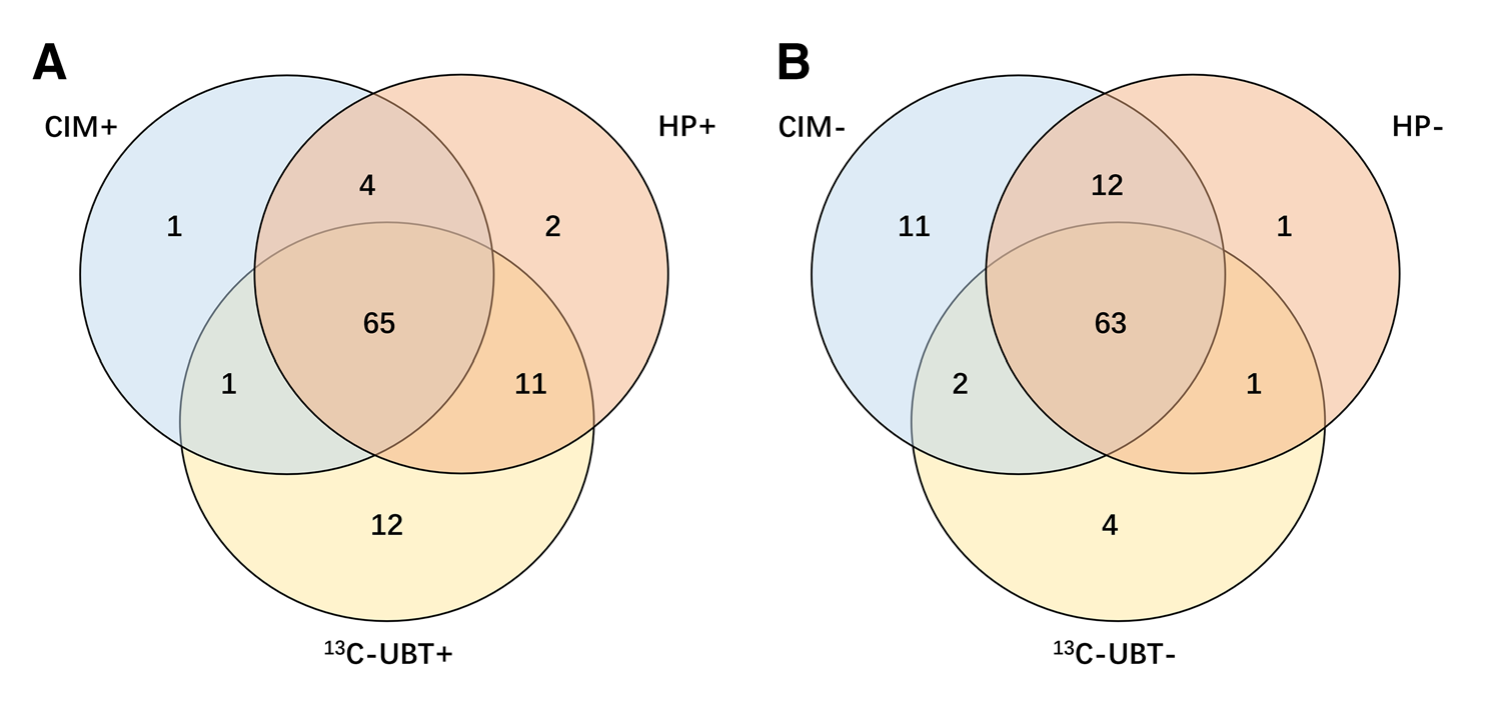
The positive and negative coincidence rates of the *H pylori* antibody serotyping test, CIM test, and ^13^C-UBT test in healthy controls and chronic gastritis groups. (A) The positive coincidence rate of the *H pylori* antibody serotyping test, CIM test, and ^13^C-UBT test; (B) The negative coincidence rate of the *H pylori* antibody serotyping test, CIM test, and ^13^C-UBT test. The positive and negative numbers of the 3 methods were marked. CIM = current infection marker, UBT = urea breath test.

The positive and negative coincidence rates of the 3 methods in the 96 subjects who were positive for any one of the 3 tests and 94 subjects who were negative for any one of the 3 tests are displayed (Fig. 2). Among the subjects, 81 subjects characterized by simultaneous positivity from 2 or 3 tests yielded a high positive coincidence rate of 84.38%, and 65 subjects exhibited simultaneous positivity with all 3 tests, resulting in a positive coincidence rate of 67.71% in reference to the 96 subjects who were positive with any one of the 3 tests (Fig. 2A). Moreover, 78 subjects displayed simultaneous negativity from 2 or 3 tests, generating a negative coincidence rate of 82.98%, and 63 subjects tested negative with all 3 tests, resulting in a negative coincidence rate of 67.02% in reference to 94 subjects who were negative with any one of 3 tests (Fig. 2B). A minority of the subjects displayed isolated positive or negative results among the 3 testing methods. These results suggested that high positive and negative coincidence rates occurred in the *H pylori* antibody serotyping, CIM, and ^13^C-UBT tests, but the positivity or negativity of the 3 tests was not completely consistent, further emphasizing the importance of combined detection with 3 tests to compensate for the deficiency of individual tests.

### 3.4. Combined detection of ^13^C-UBT and serological antibody test equivalent diagnostic efficacy as gastroscopy in diagnosing chronic gastritis

The clinical coincidence rates of simultaneous positivity and negativity among *H pylori* antibody serotyping, CIM, and ^13^C-UBT tests in patients with chronic gastritis and healthy controls are described in Table 4. Of the 65 subjects who exhibited simultaneous positivity in all 3 tests, 63 were diagnosed with type B chronic gastritis by gastroscopy, and the clinical coincidence rate reached 95.38%, which was obviously elevated compared to the individual test. Of the 63 subjects who showed simultaneous negativity in all 3 tests, 52 were healthy, and the clinical coincidence rate was 82.54%. These results indicated the advantage of combined detection with the 3 methods, characterized by high clinical coincidence rates, in diagnosing type B chronic gastritis infected with *H pylori* and exclusion of disease, and the potential to become an alternative to gastroscopy.

**Table 4:** Combined detection of *H pylori* antibody serotyping test, CIM test, and UBT test in chronic gastritis groups and healthy controls

Endoscope finding	^13^C^+^	CIM^+^	HP^+^	^13^C^+^ + CIM^+^ + HP^+^	^13^C^−^ + CIM^−^ + HP^−^
	n	n	n	n	n
CNAG group	43	31	38	32	2
CAG group	38	35	38	30	9
HC	8	5	6	3	52
Clinical coincidence rate (%)	91.01	92.95	92.68	95.38	82.54

Data were described as number and percent. ^13^C^+^ represents UBT test positive; ^13^C^−^ represents UBT test negative; CIM^+^ represents CIM test positive; CIM^−^ represents CIM test negative; HP^+^ represents *H pylori* antibody serotyping positive; HP^−^ represents *H pylori* antibody serotyping negative. The clinical coincidence rate was the rate that subjects with a positive detection index were diseased and subjects with a negative detection index were healthy.

CAG = chronic atrophic gastritis, CIM = current infection marker, CNAG = chronic non-atrophic gastritis, HC = healthy controls, HP = *H pylori*, n = number of patients.

### 3.5. A strong diagnostic value of a combination of *H pylori* antibody serotyping test, CIM test, and UBT test in type B chronic gastritis

**Figure 3.**
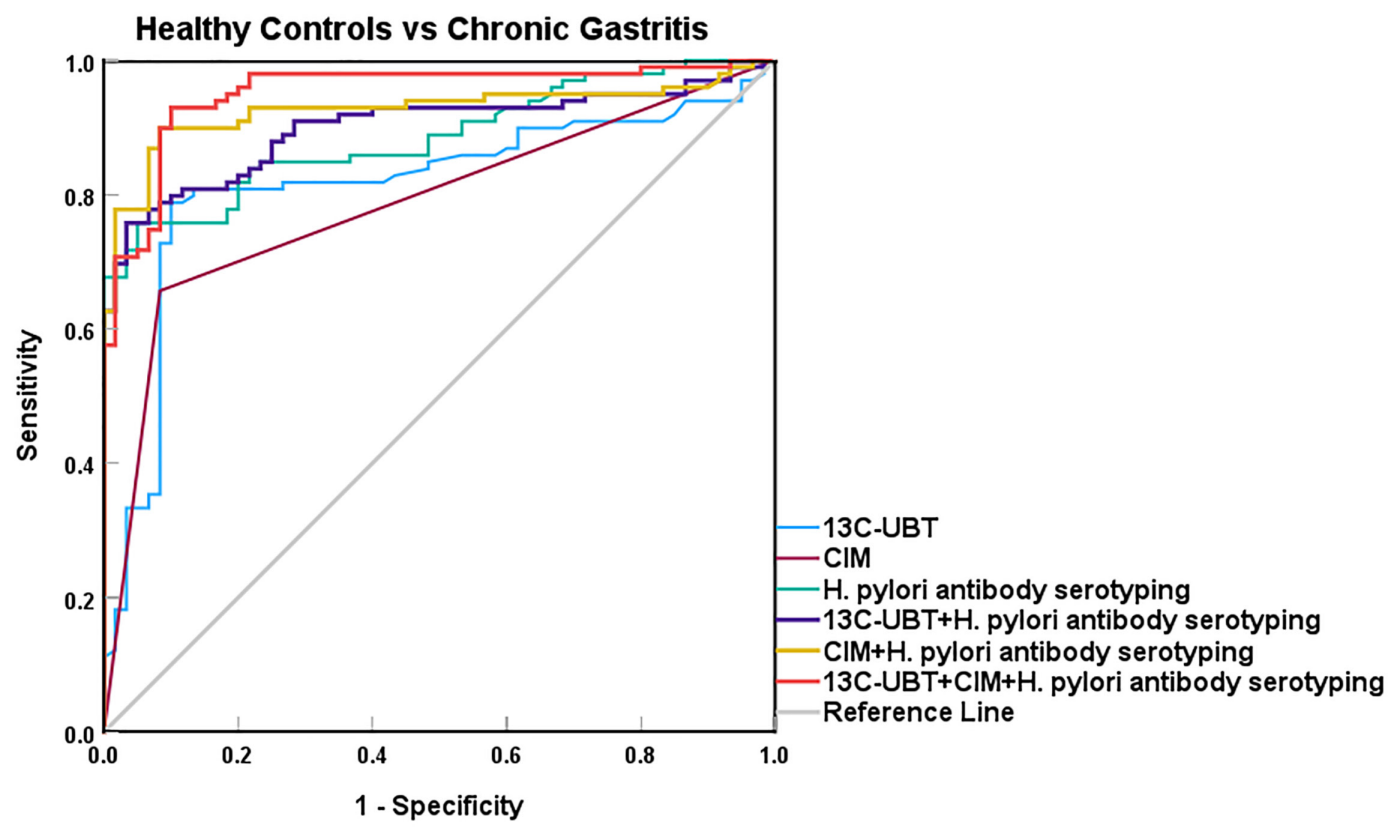
Univariate logistic regression analysis and ROC curves of ^13^C-UBT, CIM, *H pylori* antibody serotyping (anti-Urease antibody + anti-CagA antibody + anti-VacA antibody), the combination of ^13^C-UBT and *H pylori* antibody serotyping, the combination of CIM and *H pylori* antibody serotyping, and the combination of ^13^C-UBT, CIM, and *H pylori* antibody serotyping in healthy control and chronic gastritis groups. CagA = cytotoxicity-associated gene A, CIM = current infection marker, ROC = receiver operating characteristic, UBT = urea breath test, VacA = vacuolating cytotoxin A.

To evaluate the diagnostic value of *H pylori* antibody serotyping, CIM, and ^13^C-UBT for chronic gastritis, ROC curves were generated from healthy individuals and CAG or CNAG patients (Table 5) (Fig. 3). Although the AUC for the ^13^C-UBT, CIM, and *H pylori* antibody serotyping (the combined anti-Urease, anti-CagA, and anti-VacA antibodies) tests exceeded 0.5 individually (AUC = 0.815, 95% confidence interval [CI] = 0.744–0.886, *P* < .001; AUC = 0.787, 95% CI = 0.715–0.858, *P* < .001; AUC = 0.886, 95% CI = 0.836–0.936, *P* < .001, respectively), the AUC for the combination of *H pylori* antibody serotyping test, CIM test, and ^13^C-UBT test was superior to those of each test individually. Furthermore, univariate logistic regression analysis showed that the AUC for the combination of the ^13^C-UBT and *H pylori* antibody serotyping test was 0.900 (95% CI = 0.851–0.949, *P* < .001). The AUC for the combination of the CIM and *H pylori* antibody serotyping tests was even higher at 0.926 (95% CI = 0.882–0.970, *P* < .001). Additionally, the AUC for the combination of the ^13^C-UBT, CIM, and *H pylori* antibody serotyping tests was the highest among all parameters (AUC = 0.952, 95% CI = 0.919–0.985, *P* < .001), suggesting that the combined detection with the 3 tests offered a significant advantage over individual tests for the early screening and diagnosis of CNAG and CAG associated with *H pylori* infection.

**Table 5:** Diagnostic value of combined detection with *H pylori* antibody serotyping test, CIM test, and UBT test for healthy controls and chronic gastritis.

Parameters	AUC	95% CI	*P* value
^13^C-UBT	0.815	0.744–0.886	< .001
CIM	0.787	0.715–0.858	< .001
*H pylori* antibody serotyping	0.886	0.836–0.936	< .001
^13^C-UBT + *H pylori* antibody serotyping	0.900	0.851–0.949	< .001
CIM + *H pylori* antibody serotyping	0.926	0.882–0.970	< .001
^13^C-UBT + CIM + *H pylori* antibody serotyping	0.952	0.919–0.985	< .001

Univariate logistic regression analysis of ^13^C-UBT, CIM, *H pylori* antibody serotyping (the combination of anti-Urease, anti-CagA, and anti-VacA antibodies), the combination of ^13^C-UBT and *H pylori* antibody serotyping, the combination of CIM and *H pylori* antibody serotyping, and the combination of ^13^C-UBT, CIM, and *H pylori* antibody serotyping in healthy controls and chronic gastritis groups was conducted using SPSS software (31.0). The AUC, 95% CI, and *P* value were analyzed and calculated.

AUC = area under the curve, CagA = cytotoxicity-associated gene A, CI = confidence interval, CIM = current infection marker, n = number of patients, UBT = urea breath test, VacA = vacuolating cytotoxin A.

## 4. Discussion

The incidence of *H pylori* infection varies significantly across regions. In developed countries, this incidence is declining owing to improved sanitary conditions and the extensive use of antibiotics.^[6]^ *H pylori* infection is the primary cause of type B chronic gastritis, and its prevalence is generally consistent with the rate of *H pylori* infection and may be slightly higher than that of *H pylori* infection in some cases.^[11,12]^ Gastroduodenal disease induced by *H pylori* infection involves a series of inflammatory responses, including activation of pro-inflammatory cytokines and overexpression of vascular endothelial growth factor. These responses are influenced by factors such as the type of *H pylori* strain, host genetics, immune functions, and dietary habits. Therefore, the early diagnosis of *H pylori* infection is crucial to prevent the development of gastroduodenal diseases.^[5,13]^

The principal virulence factors of *H pylori* are CagA and VacA. CagA, in particular, serves as a crucial determinant of the pathogenicity of *H pylori* strains.^[2,14]^ CagA is released into gastric epithelial cells after *H pylori* infection, where it interacts with various host proteins that regulate essential cellular functions. VacA can trigger proapoptotic and pro-inflammatory responses that are implicated in peptic ulceration.^[6,15]^ *H pylori* strains are classified as type I *H pylori* (CagA+/VacA+) and type II *H pylori* (CagA−/VacA−) based on the expression of anti-CagA and anti-VacA antibodies. Accumulating evidence suggests that type I *H pylori* infection, which is characterized by higher virulence factors and greater migratory ability, is more likely to result in gastric mucosal atrophy. In contrast, type II *H pylori* infection is associated with weaker progression of atrophy.^[2,16]^ Consistent with these findings, our study revealed that type I *H pylori* infection was more prevalent in the CNAG and CAG groups than in the type II group.

The early stages of type B chronic gastritis pose challenges for detection, and multiple methods must be employed to accurately identify active *H pylori* infection.^[11,17]^ Our results showed significantly elevated levels of anti-urease antibody, anti-CagA antibody, anti-VacA antibody, and ^13^C-UBT in the CNAG and CAG groups compared to those in the control group, especially in the CNAG group. Although the results of the *H pylori* serotyping antibody test and ^13^C-UBT were not entirely synchronized, a significant positive correlation was observed between these markers. A previous study by Fei Wang reported high positivity rates for *H pylori* serotyping antibodies in chronic gastritis, aligning with our findings of markedly increased positive rates for anti-CIM IgG and *H pylori* serotyping antibodies in the CNAG and CAG groups compared to controls, with the CAG group exhibiting a higher positive rate.^[10]^ Therefore, these studies suggest that the *H pylori* antibody serotyping test, serological CIM test, and ^13^C-UBT test can play a crucial role in the early screening and diagnosis of type B chronic gastritis.

Previous research has reported high positive and negative coincidence rates between the CIM and ^13^C-UBT tests, which is consistent with our findings.^[8]^ Our results showed high positive coincidence rates between the *H pylori* antibody serotyping test or CIM test and the ^13^C-UBT test in the separate comparison, which was prominent, especially in type I *H pylori* serotyping. Furthermore, several studies have noted that the ^13^C-UBT may be limited in patients who have recently taken antibiotics or proton pump inhibitors, which may have a weak impact on the *H pylori* antibody serotyping test and the CIM test.^[8,18]^ Consequently, the combined application of the 3 tests can reduce disturbance and enhance diagnostic accuracy for *H pylori* infection, further emphasizing the importance of combined detection with several methods to compensate for the deficiency of individual tests. In our study, isolated positive or negative results of the 3 tests occurred, whereas simultaneous positive and negative results from 2 or 3 tests generated a higher positive coincidence rate of 84.38% and a negative coincidence rate of 82.98% when compared to any one of the 3 tests, positive or negative, in all subjects. In addition, the clinical coincidence rates between endoscopic findings and simultaneous positivity with 3 tests were researched; 95.38% of subjects who tested positive simultaneously with 3 tests were diagnosed with chronic gastritis by gastroscopy, while 82.54% of subjects who were characterized by simultaneous negativity with 3 tests were healthy, indicating a high clinical coincidence rate of combined detection with 3 tests, which was higher than each individual test. These results accounted for the high consistency among the 3 tests in diagnosing type B chronic gastritis infected with *H pylori* and demonstrated the advantage of combined detection in the diagnosis and exclusion of disease.

We further investigated the diagnostic value of these 3 tests, both individually and in combination. Our findings demonstrated that *H pylori* antibody serotyping, CIM, and ^13^C-UBT exhibited high sensitivity and specificity for diagnosing chronic gastritis. Notably, the diagnostic value was significantly enhanced when the 2 tests were combined compared to any single test. Furthermore, the combined detection of the *H pylori* antibody serotyping test, CIM test, and ^13^C-UBT generated a markedly higher predictive and diagnostic value for type B chronic gastritis, distinguishing healthy controls. The diagnostic sensitivity and specificity of the combined approach with 3 tests exceeded those of any individual test or any combination of 2 tests, with an AUC of ROC reaching 0.952. Our results suggest that the combined detection of *H pylori* antibody serotyping, CIM, and ^13^C-UBT tests may hold greater diagnostic significance than any single test alone and can serve as a noninvasive early screening diagnostic indicator for CNAG and CAG, potentially contributing to improved clinical prevention and treatment in the early stages. Additionally, our limitations are that the simultaneous negative results of the 3 tests largely exclude *H pylori* infection, but this does not completely rule out the occurrence of CNAG and CAG, which may arise from other factors.

## 5. Conclusion

*H pylori* antibody serotyping, CIM, and ^13^C-UBT play important roles in the screening and diagnosis of chronic gastritis associated with *H pylori* infection, and they have distinct advantages and limitations, respectively. Combined detection with 3 tests may have greater predictive and diagnostic value than any single test alone, which significantly improves diagnostic sensitivity and accuracy and enhances early screening diagnostic potential for CNAG and CAG. This noninvasive approach shows promise for supporting clinical prevention and early treatment strategies.

## Acknowledgements

This work was supported by grants from the Knowledge Innovation Project of Wuhan Science and Technology Bureau (No. 2023020201010184) and Provincial Health Commission Research Project (No. WJ2023F042). We would like to thank the Wuhan Municipal Health Youth Talent Training Program.

## Author contributions

**Conceptualization:** Yin Xiong, Beibei Wang, Ping Wu, Wei Huang.

**Data curation:** Yin Xiong.

**Methodology:** Ke Li.

**Software:** Xin Chen.

**Validation:** Ping Wu.

**Writing – original draft:** Yin Xiong.

**Writing – review & editing:** Beibei Wang, Wei Huang.
